# Oral squamous cell carcinoma in non-smoking and non-drinking patients

**DOI:** 10.1186/1758-3284-2-24

**Published:** 2010-10-04

**Authors:** Astrid L Kruse, Marius Bredell, Klaus W Grätz

**Affiliations:** 1 University Hospital Zurich, Department of Craniomaxillofacial and Oral Surgery, Zurich/Switzerland

## Abstract

**Introduction:**

Of the many different factors associated with an increased risk for oral squamous cell carcinoma (SCC), tobacco and alcohol seem to be the most studied. The aim of the current study was to evaluate the clinicopathologic characteristics of patients without the mentioned risk factors.

**Patients and Methods:**

Out of 278 patients (159 male and 119 female patients) with oral SCC, 67 patients had no history of tobacco or alcohol use. The minimum follow-up time was 12 months.

**Results:**

Of the 67 patients, 45 (67.2%) were women, and the mean age was 70 years. The most common tumor sites were mandibular alveolar ridge (22) and maxilla (18). Fifteen patients experienced a recurrence, and 10 developed lymph node metastases during the follow-up period.

**Conclusion:**

The group of patients with no tobacco and alcohol use tends toward a higher proportion of females, a higher proportion of patients over 70 years, and a higher number of oral maxillary SCC.

## Introduction

Of the many different factors associated with an increased risk for oral squamous cell carcinoma (SCC), tobacco and alcohol seem to be the most studied. Individuals who smoke more than 20 cigarettes a day and consume more than 100 g of alcohol a day are at increased risk for oral epithelial dysplasia, but ex-smokers of 10 or more years seem to have no greater risk than non-smokers [1]. In addition, alcohol has been found to be an independent risk factor for oral SCC among non-smokers [2] and tobacco smoke in non-drinkers [3,4]. The combination of both factors seems to enhance the carcinogenic effect [5,6]. Blot et al. (1988) [6] stated that tobacco smoking and alcohol drinking combine to account for approximately three-fourths of all oral and pharyngeal cancers in the United States. However, the other fourth of patients are then of special interest, because this group has rarely been studied. Regarding lung cancer in patients without tobacco use, some authors have stated that these cancers are their own entity due to their special characteristics.

Therefore, the aim of the current study was to evaluate the clinicopathologic characteristics of the patient group without the tobacco and alcohol risk factors, in particular concerning gender, location, TN status, and rates of metastases or recurrence.

## Patients and Methods

The files of 278 patients (159 male and 119 female patients) with newly diagnosed, previously untreated oral SCC and who were treated between 1999 and 2008, with a minimum follow-up time of 12 months, were searched for patients without the risk factors of tobacco or alcohol use. Out of these 67 patients, tumor data--including site, grade, TN status, recurrence, and metastases--were obtained from a review of the medical records.

## Results

**Figure 1:**
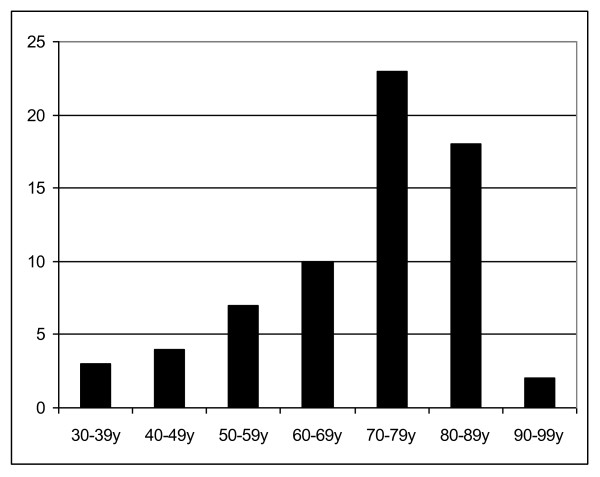
**Age distribution**.

In sum, 22 male and 45 female patients were without the tobacco or alcohol risk factors. Of these 67 patients, 43 (64.2%) patients were over 70 years and only 3 (6%) below the age of 40. The mean age was 70 years (Fig. 1).

**Figure 2:**
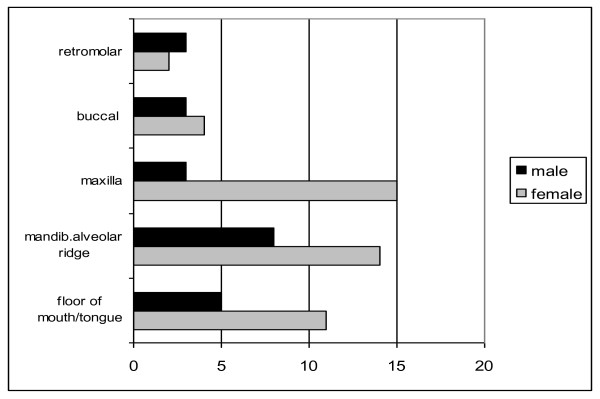
**Distribution of tumor location of patients without known risk factors (tobacco/alcohol)**.

The most common tumor sites were the mandibular alveolar ridge (22 patients) and the maxilla (18 patients). The most frequent sites in female patients were oral maxillary SCC (15/67), followed by the mandibular alveolar ridge (16/67) (Fig. 2).

The distribution of T and N status is shown in Table 1. T1 and T2 tumors were present in 48 out of 67 cases. Of the 67 patients, 47 had no primary lymph node metastases. Thirty-one patients out of 67 revealed a median differentiated SCC, followed by well differentiated in 20 cases. Of the 67 patients, 15 developed recurrence, 10 metastasis, and 3 both conditions during a median follow-up time of 16.7 months (minimum 12 months).

**Table 1:** Patients' characteristics

Characteristics	Number of patients
*T status*	
T1	24
T2	16
T3	3
T4	24

*N status*	
N0	47
N1	11
N2a	1
N2b	5
N2c	3

*Grading*	
1	20
2	31
3	16

*Recurrence*	15

*Metastasis*	10

*Metastasis and recurrence*	3

## Discussion

Data from several reports [7] indicate that the exposure of women to both tobacco and alcohol risk factors causes a change in the male to female ratio in favor of women for oral tumors. But the present findings differ from the previously recorded data. The gender distribution of 45 female to 22 male patients in the present study was striking. But the discovery of female predominance in this patient group is also supported by other studies [8-10]. Concerning the distribution, Dahlstrom et al. (2008) [9] reported that mostly young women with oral tongue cancer, elderly women with gingival/buccal cancer or young to middle-aged men with oropharyngeal cancer belong to the oral SCC patient group that does not have the risk factors of tobacco and alcohol; Harris et al. (2010) [8], on the other hand, stated that mainly young patients are involved. Our data are similar to the results of Schmidt et al. (2004) [11], who reported a higher average age of non-smokers in comparison to smokers (71.4 versus 63.7 years).

Regarding the site of tumor presentation, Schmidt et al. (2004) [11] reported a significant association with smoking and the posterolateral tongue and floor of mouth sites. This finding can be supported by the present results with the main distribution in the mandibular alveolar ridge and maxilla in the group of patients without tobacco or alcohol. One reason for fewer tumors of the tongue or floor of mouth in comparison to the oral maxilla could be that the floor of the mouth and tongue are particularly sensitive to the carcinogenic effects of tobacco or alcohol [11,12]. The relatively high number of oral maxillary SCC in elderly patients has also been reported before [13].

However, the etiology of oral SCC in those who have never smoked is still unclear. A viral association, particularly the human papilloma virus (HPV), has been implicated in the pathogenesis. The prevalence of HPV, mainly HPV 16, is high in oropharyngeal SCC [14], but concerning the prevalence of HPV in the oral cavity--mostly tongue SCC as a main location for oral cavity cancer--the data from the literature are not concordant, ranging from 2.6 to 98% [15,16].

The question of differences between smokers and non-smokers seems to play a role not only in oral SCC, but also in lung SCC [17], suggesting that different characteristics like mutations in the p53 and K-ras genes [18], HPV 16/18 infections [19], or outcome after treatment with epidermal growth factor receptor tyrosind kinase (EGFR-TH) inhibitors lead to a unique entity in non-smokers.

Concerning the outcome, 15 patients experienced a recurrence, 10 developed lymph node metastases, and 3 both conditions during the follow-up period; these results are similar to those of Wisemand et al. (2003). Relative to distribution of TN status, recurrence and metastases rates seem to be no different.

In conclusion, the group of patients with no tobacco and alcohol use tends toward a higher proportion of females, a higher proportion of patients over 70 years, and a higher number of oral maxillary SCC.

## Competing interests

The authors declare that they have no competing interests.

## Authors' contributions

AK carried out the retrospective study, MB drafted the manuscript and KG participated in the design of the study and coordination. All authors read and approved the final manuscript.
